# Supplementary material for: A stem cell population at the anorectal junction maintains homeostasis and participates in tissue regeneration
Source: Nat Commun. 2021 May 12;12:2761. doi: 10.1038/s41467-021-23034-x (PMC8115161; doi:10.1038/s41467-021-23034-x)
Supplement: Supplementary file 1 — Supplementary Information [file 41467_2021_23034_MOESM1_ESM.pdf]

Supplementary information

A stem cell population at the anorectal junction maintains homeostasis and participates in tissue regeneration

Louciné Mitoyan[1], Véronique Chevrier[1], Hector Hernandez-Vargas[2,3], Alexane Ollivier[1], Zeinab Homayed[4], Julie Pannequin[4], Flora Poizat[5], Cécile De Biasi-Cador[5], Emmanuelle Charafe-Jauffret[1,5], Christophe Ginestier[1], and Géraldine Guasch[1]*

Supplementary Figure 1. Active renewal occurring in the anorectal transition zone.

a. Immunofluorescence staining shows the squamous nature of the anal canal and the TZ expressing the Krt14 (red) and the glandular nature of the rectum expressing the Krt8 (white) (n=4 independent experiments from 4 mice). **b.** Krt17 (white) is strongly expressed at the TZ and weakly in anal canal cells proximal to the TZ. Section is from mouse anorectal day 31 oil control (n=22 independent experiments from 25 mice). **c.** Proliferative cells are detected at the TZ at a higher rate compared to the anal canal. Quantification of the % of Edu positive cells in TZ and anal canal area (Source data are provided as a source data file). EdU+ cells were quantified based on differential CD34 expression found in the TZ compared to the anal canal as previously described[11,38]. At least 9 different areas were analyzed for TZ and anal canal for each mouse (n=5). Total number of cells was counted using DAPI staining (reported as 100%) in TZ and anal canal. Paired t test two-tailed; error bars, mean ± SEM ***$p<0.0001$. Representative image of Edu positive cells (nuclear white staining) at the TZ and in the anal canal area. Source data for the Edu quantification is provided as a Source Data file**. d.** Unipotency of Krt17+ TZ cells (green) during long-term lineage tracing (n=3 mice) (left image) and representative images of n=3 biologically independent samples of oil control injected mice

analyzed after two days of chase and stained with Krt17 in white and Krt8 in red. Dashed lines delineate the epithelium from the stroma. Scale bars are 50 μm.

Mitoyan et al., Supplementary Figure 1

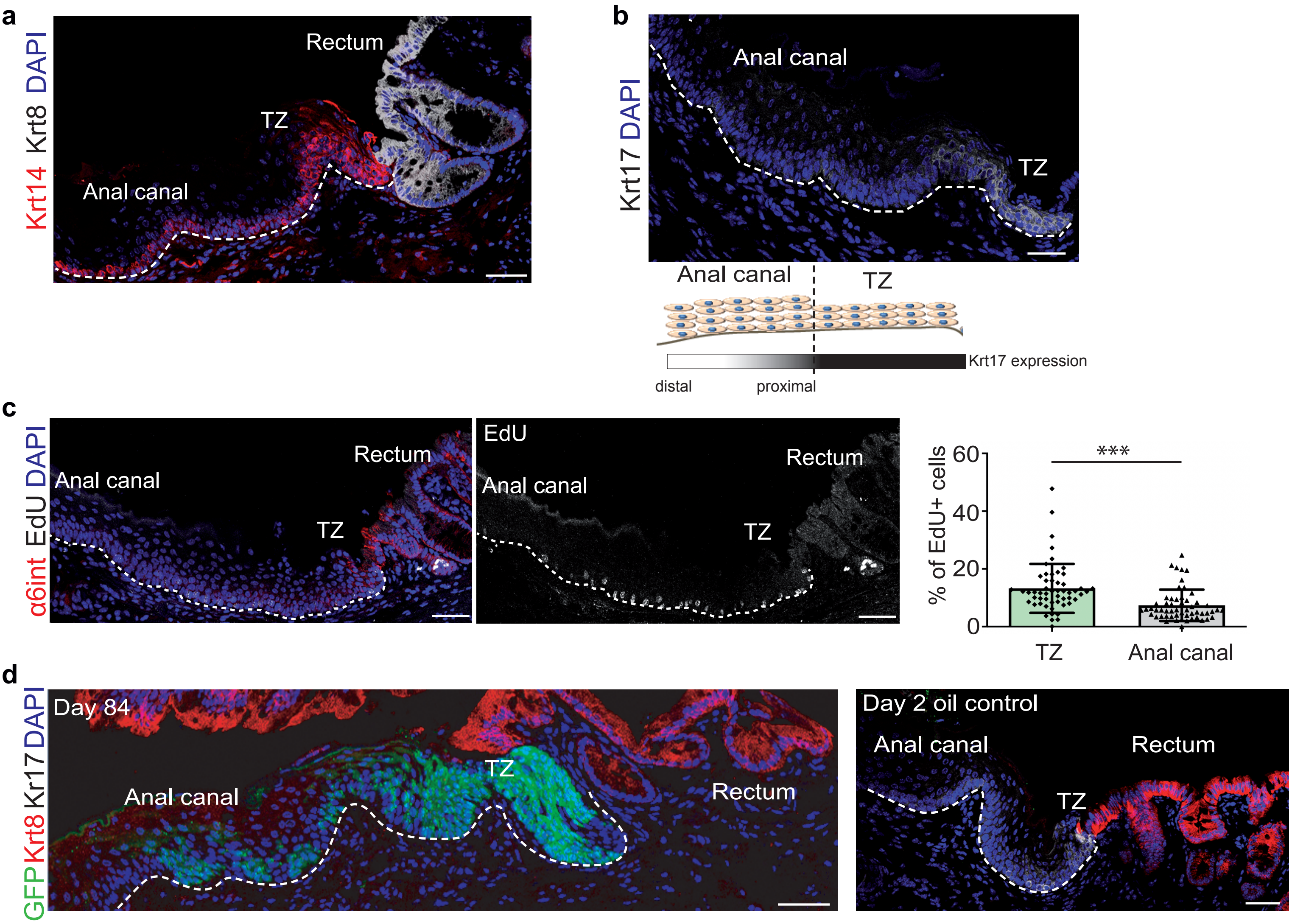

Supplementary Figure 2. Expression of *Krt17* transcript in the anorectal region.

a,b. high quality in-situ hybridization (RNAScope) shows expression of *Krt17* in the anal TZ. Representative images of n=3 biologically independent samples in which fluorescence has been quantified in the absence or presence of 2 days tamoxifen injection. Insets are zoom in 1.2 fold (AC RNA), 1.4 fold (TZ RNA), 1.3 fold (AC protein), 1.25 fold (TZ protein). **b.** Positive control shows expression of *Actin* transcript in all tissue and **c.** negative control shows background level. Insets are zoom in 1.42 fold (AC and TZ RNA positive control) and1.6 fold (AC and TZ RNA negative control) **c.** Normalized dot/ROI *Krt17* RNA expression visualized by RNAscope technology (see supplemental methods for details and source data are provided as a source data file). (n=3 mice were analyzed per condition). Unpaired t test two-tailed error bars, mean ± SEM **p=0.0011 ***p<0.0001. At least 6 areas of each region (anal canal, TZ and rectum) were quantified per mice (n=3 mice were quantified per condition). Source data for the *Krt17* RNA quantification is provided as a Source Data file**.** Scale bars are 50µm.

Mitoyan et al., Supplementary Figure 2.

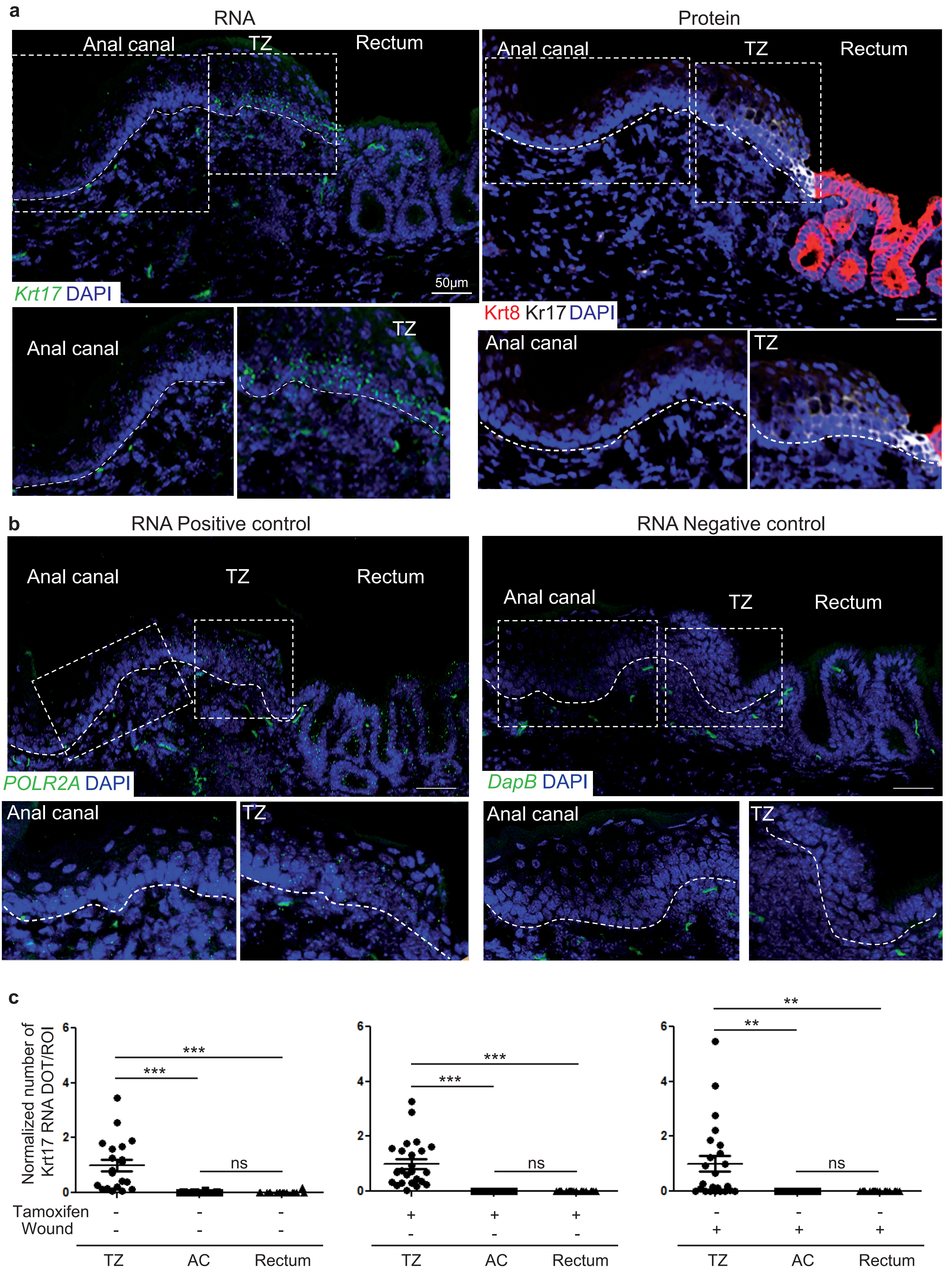

Supplementary Figure 3. Tamoxifen injection does not induce a wound response. a. Tamoxifen does not induce Krt17 expression in the anal canal and in rectum. Krt17 protein integrated density was measured by ImageJ in unwound conditions in the presence and absence of tamoxifen injected twice and 48h post-wound without tamoxifen in transition zone, anal canal (**ai**) and rectum (**aii**). Unpaired t test two-tailed $*p \leq 0.0376$ **b.** Quantification of EdU expression shows that tamoxifen injection does not change the proliferation status of Krt17+ TZ cells. At 48h post-wound, rectal cells are highly proliferative compared to TZ. Representative images of Edu positive cells (nuclear white staining at the TZ and rectum) **c-d.** Quantification of CD34 and EdU expression shows that tamoxifen injection does not change either the proliferation status of CD34+ TZ cells or the CD34 expression. Note that upon wounding, CD34 is expressed in the anal canal cells. Unpaired t test two-tailed $*p=0,0256$ $**p \leq 0,0039$ $***p \leq 0,0006$. Quantifications for **a**, **b**, **c** and **d** were done using at least 8 areas of each region (anal canal, TZ and rectum) per mice (n=3 mice were quantified per condition). (Source data are provided as a source data file).

Mitoyan et al., Supplementary Figure 3.

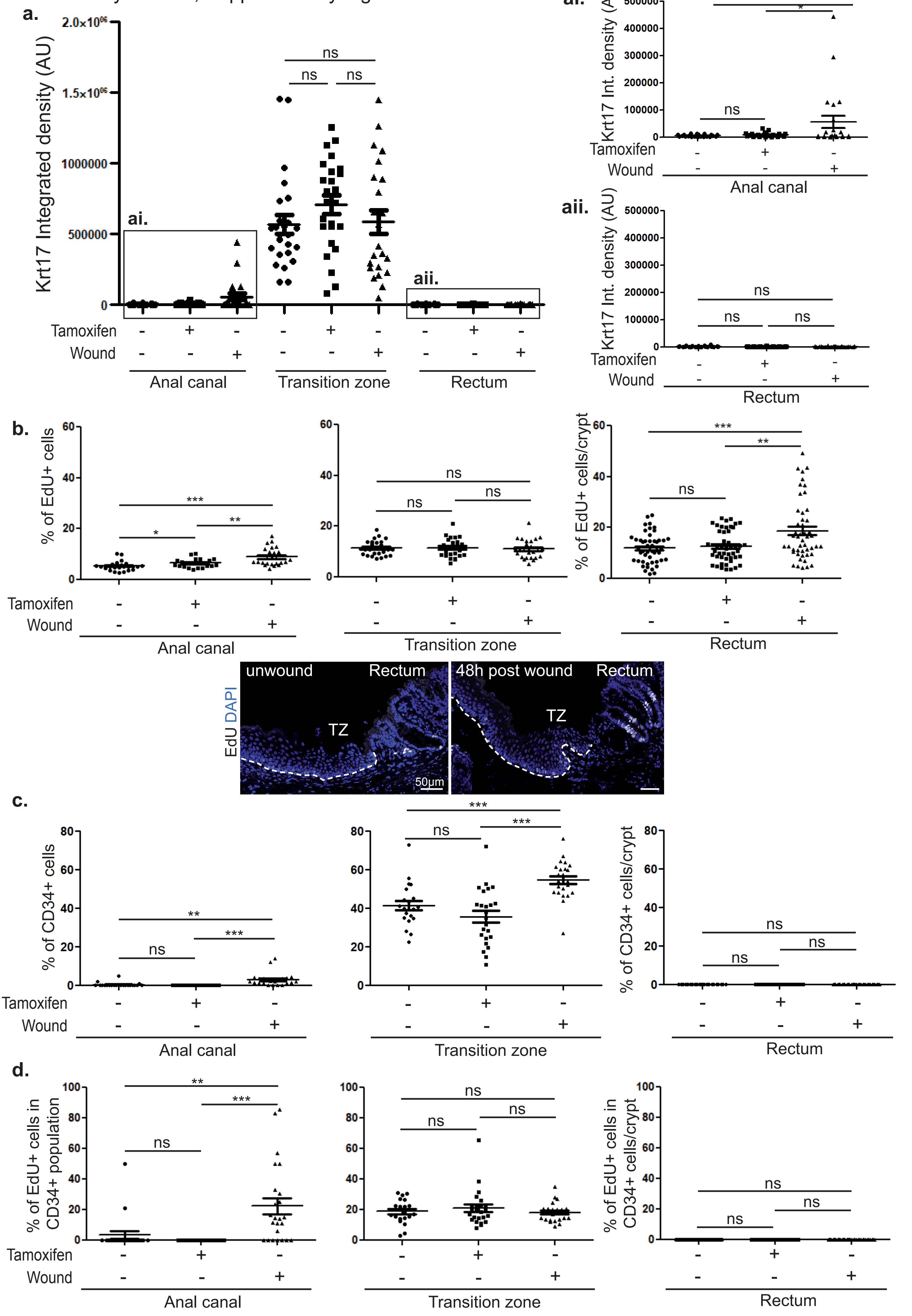

Supplementary Figure 4. Strategy to isolate the anorectal region

Representative example of the FACS strategy to isolate TZ cells, anal canal and rectum from *K17CreER*^{T2}*;R26R*^{GFP} mice induced with tamoxifen for 2 days (n=20). CD45+ blood cells, CD11b+ macrophages, CD31+ endothelial cells and PDGFRα+ fibroblasts were excluded from the live (FVD-780-), K17+GFP+ population. These epithelial cells were further purified by gating for EpCam+ cells. Anal canal cells are Epcam+GFP- and rectum cells are Epcam^{High} GFP-. An example of the purity of each population is described.

Mitoyan et al., Supplementary Figure 4

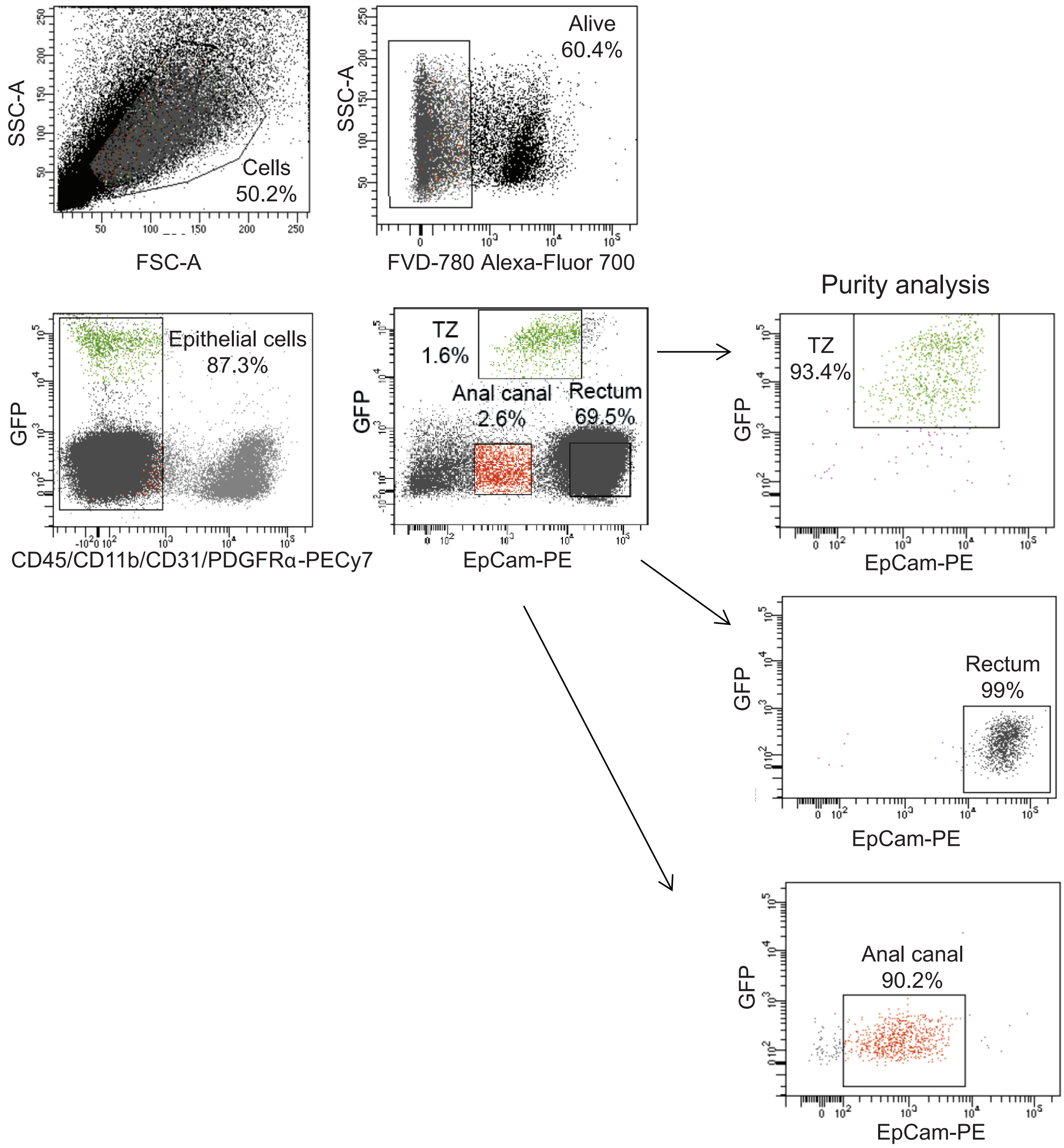

Supplementary Figure 5. Single-Cell RNA seq of FACS-sorted anorectal cells

a. Main cell populations visualized on Uniform Manifold Approximation and Projection (UMAP) dimensional reduction from scRNA sequencing of anorectal epithelial cells. **b.** To determine the pathways associated with the cluster 1, we perform a gene functional classification using DAVID algorithm. Bar plot represents enrichment for each of the pathway identified, where the strength of the association is represented by the log10 (p-value). A majority of genes defining cluster 1 appears to be involved in lipid synthesis, a characteristic of the anal gland (Source data are provided as a source data file). A two-sided Fisher's exact test was used for data analysis. **c.** Immunofluorescence with perilipin-2 antibody (red), found in cluster 1, confirms that it represents the anal gland populations positive for oil Red O (n=3 independent experiments from n=3 mice). Scale bars are 50 µm for the immunofluorescence and 20 µm for the oil Red O staining. **d-e.** Expression of key genes expressed in the cluster 7 representing the fibroblast **(d)** and the cluster 3 representing the hair follicle (**e**). Source data for the genes in clusters 1, 3 and 7 is provided as a Source Data file**.** Darker color in the UMAP plot indicates higher expression level of the selected gene. **f.** Immunofluorescence of Lhx2 that specifically marks the hair follicle but is absent in TZ (n=4 independent experiments from n=3 mice), confirms the hair follicle nature of cluster 3. **g.** RNAscope shows expression of *Lgr5* in the hair follicle bulge and at the base of the rectal crypt but not in the anal TZ (n=3 independent experiments from n=3 mice).

Mitoyan et al., Supplementary Figure 5

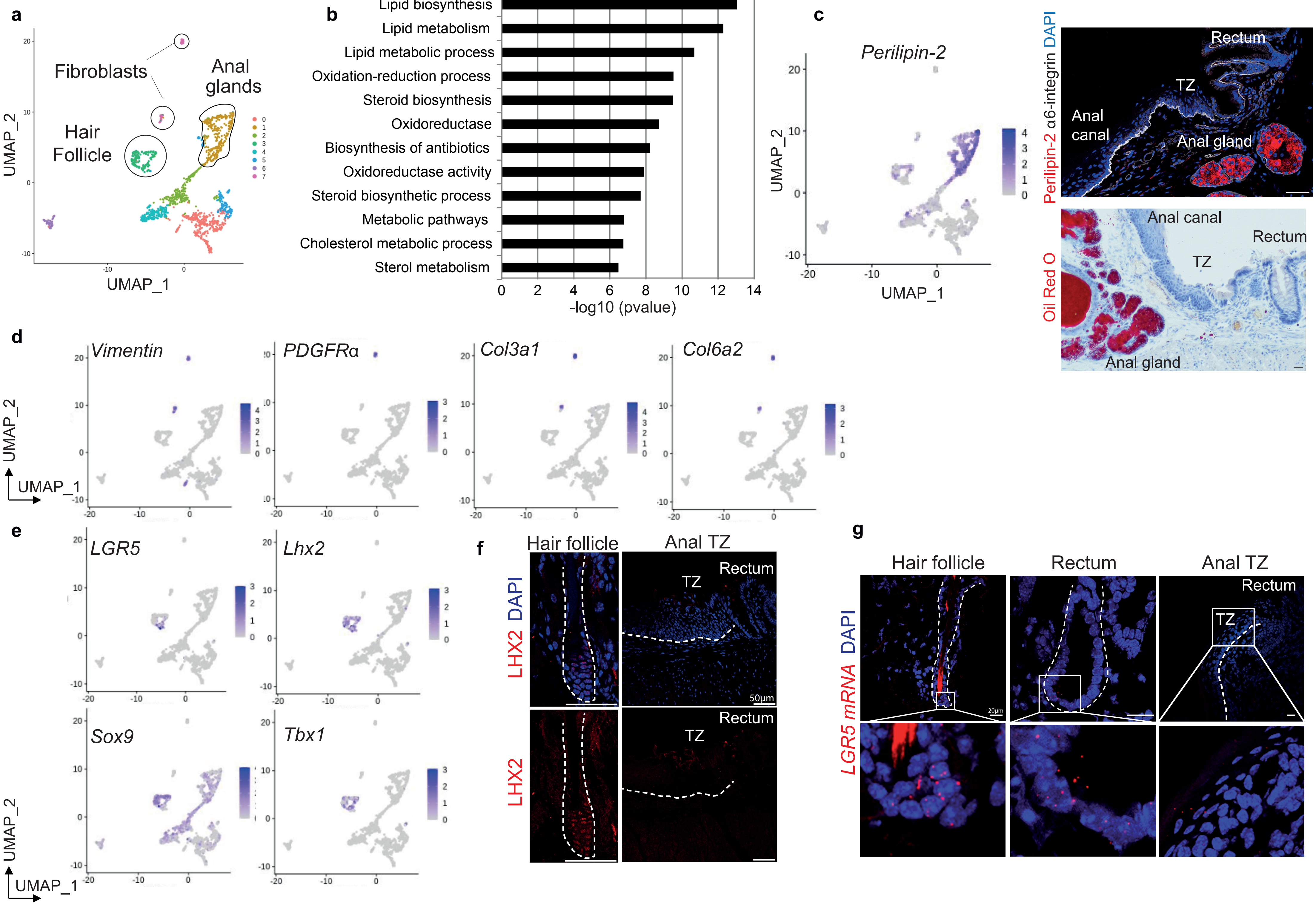

Supplementary Figure 6. Markers of the anal canal and TZ.

a. Tamoxifen induces GFP expression specifically in transition zone-derived organoids. Anal canal cells were sorted by previously established FACS strategy. As a positive control, $Epcam^{low}$ cells from non-induced $K17CreER^{T2}$;$R26R^{GFP}$ mice were sorted. At passage 1, culture media was supplemented with tamoxifen or oil control during 7 days. Brightfield and GFP images were acquired at day 7 with the Leica M205FA stereomicroscope and showed the specific induction of GFP expression in transition zone organoids in the presence of Tamoxifen compared to oil control condition and anal canal-derived organoids (n=3 independent experiments from 3 independent FACS sortings). **b.** Subcloning strategy to test the self-renewal potential of anal TZ cells compared to the anal canal and rectal cells. At passage 0, organoids were individually isolated under a Leica stereomicroscope M205FA, dissociated into single cells and replated in new matrigel (P1). This process was repeated as long as organoids grew. **c.** Cells were sorted according to previous established FACS strategy but adding Nectin antibody here. After stroma exclusion, epithelial cells were purified by gating for $EpCam^{low}GFP+$ cells. These epithelial cells were further purified by gating for Nectin4+ and Nectin4- cells. **d.** Clonogenicity assay shows that TZ basal organoids have unlimited clonogenic property whereas TZ suprabasal organoids cannot survive passage 2 (n=2).

Mitoyan et al., Supplementary Figure 6

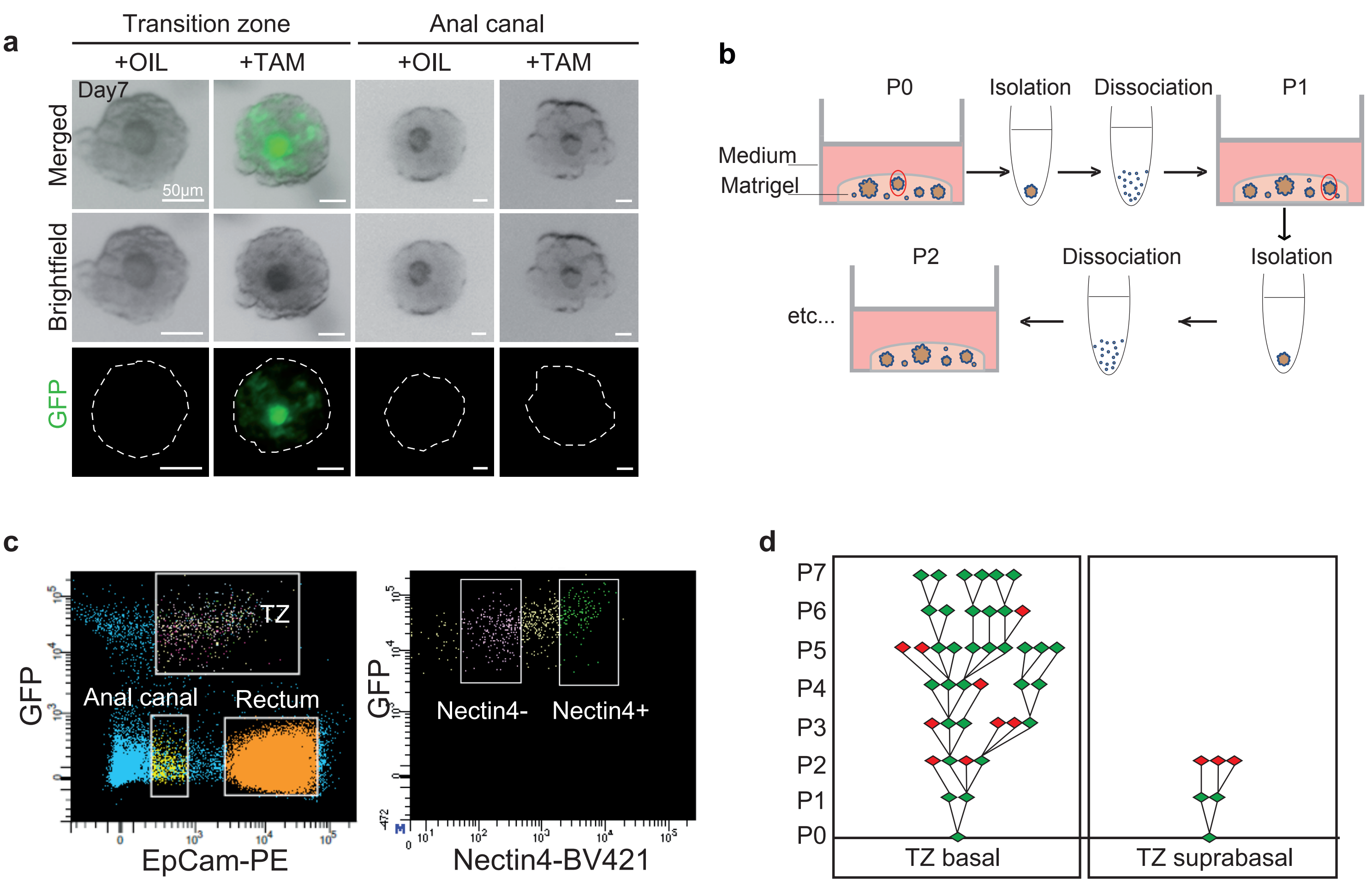

Supplementary Figure 7. Mechanical wound at the anal TZ shows multipotency of anal TZ

a. Wound strategy in *K17CreER*^{T2}*;R26R*^{GFP} bigenic mice induced with tamoxifen 2 days before injury. **b.** HES at day 0 shows wounded region in the rectum epithelium close to the TZ (n=3 mice analyzed). **c.** Similar to the EDTA wound, Few TZ-derived GFP+ cells were found in the rectum epithelium in a transition state co-expressing the glandular marker Krt8 (red) and the TZ marker Krt17 (white) (n=5/13 mice analyzed) Insets are zoom in 4 fold (left panel) and 2.5 fold (right panel). TZ cells give rise to differentiated goblet cells (alcian blue+) (n=3 independent experiments). GFP+ TZ cells participating in the regenerative crypt after a mechanical wound are JunB+ (white) and CD34+ (red) (n=3 independent experiments). Scale bars are 250 µm for **b** and 50µm for **c.**

Mitoyan et al., Supplementary Figure 7

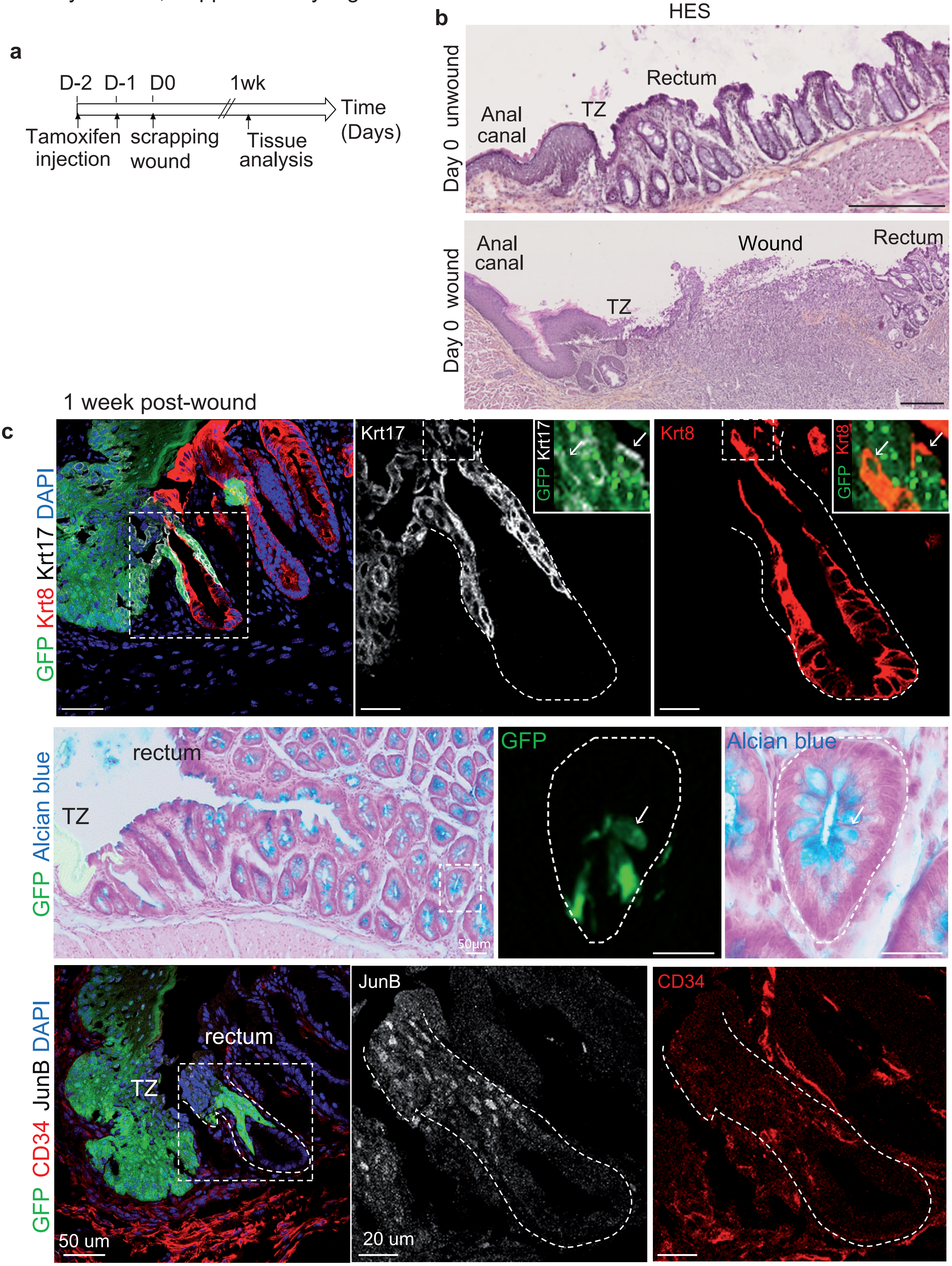

Supplementary Figure 8. Analysis of the single-Cell RNA seq of FACS-sorted anorectal cells 1 week post-EDTA wound

a-b. Representative example of the FACS strategy to isolate TZ cells, anal canal and rectum from *K17CreER*^{T2}*;R26R*^{GFP} mice induced with tamoxifen for 2 days, wounded with EDTA and analyzed 1 week later. **b.** Epcam expression profile is maintained at 1 week post EDTA wound in stratified and glandular epithelia (n=3 independent experiments from n=3 mice). **c.** Main cell populations visualized on Uniform Manifold Approximation and Projection (UMAP) dimensional reduction from scRNA sequencing of anorectal wounded epithelial cells. (Source data are provided as a source data file). **d.** Gene functional classification using DAVID algorithm was used to reveal lipid synthesis-associated pathways in cluster 2 which characterizes anal gland cluster. A two-sided Fisher's exact test was used for data analysis. Source data for the genes in clusters 2, 7 and 8 is provided as a Source Data file**. e.** Expression of key genes expressed in the different populations of wounded epithelial anorectal cells. Darker color in the UMAP plot indicates higher expression level of the selected gene. **f.** Clustering analysis showing the top genes significantly associated with the differentiation paths of trajectories 2 and 3. Heatmaps represent normalized gene expression in logarithmic scale.

Mitoyan et al., Supplementary Figure 8

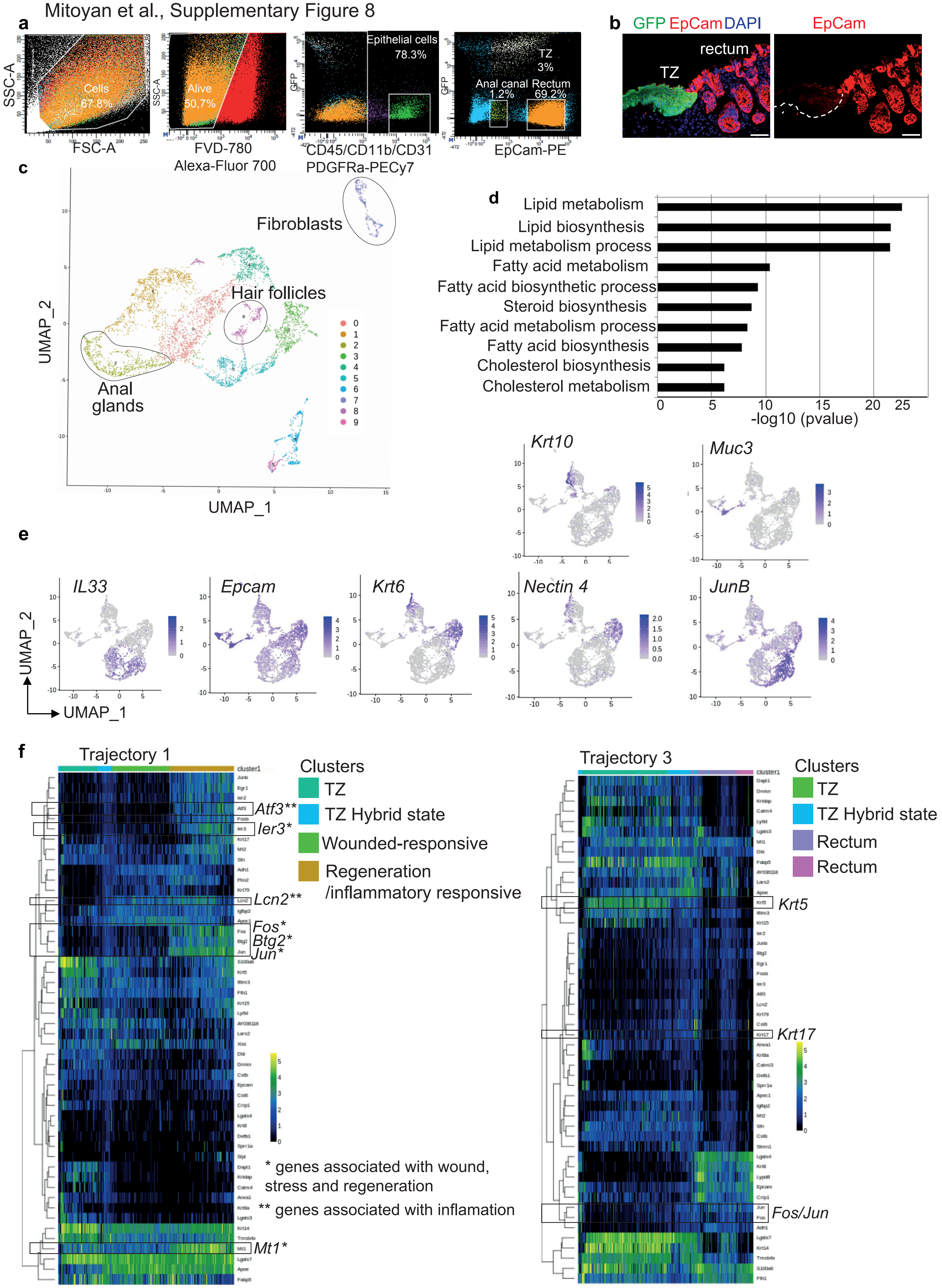

Supplementary Table 1 – Antibodies

Primary Antibody	Host Species	Supplier	Catalogue number	Dilution
α6-integrin	Rat	BD Biosciences	555734	1/100
SP-1 Chromogranin A	Rabbit	Euromedex	20086-IMMUNO	1/200
PE-EpCam<br>CD326	Rat	Biolegend	118206	1/100
Biotin-CD34	Rat	eBioscience	13-0341	1/50
Gpc3	Mouse	Thermofischer	MA5-17083	1/1000
JunB	Rabbit	Cell Signaling	3753	1/500
Keratin8	Rat	Developmental Studies Hybridoma Bank (DSHB)	AB_531826	1/50
Keratin8	Rat	Millipore	MABT329-25UG	1/400
Keratin10	Rabbit	OZYME BIOLEGEND	905401	1/1000
Keratin10	Rabbit	OZYME BIOLEGEND	905404	1/500
Keratin14	Mouse	Millipore	MAB3232	1/400
Keratin17	Rabbit	Abcam	Ab53707	1/20000
Keratin 6	Rabbit	Biolegend	905701	1/500
Lhx2	Rabbit	Millipore	ABE1402	1/2000
Loricrin	Rabbit	OZYME BIOLEGEND	905101	1/500

Lysozyme	Rabbit	DAKO	A0099	1/500
Nectin-4 clone N4.mu1	Mouse	Dr. Marc Lopez's gift	NA	1/200
PE-Cy7-CD11b	Rat	BD Pharmingen	561098	1/200
PE-Cy7-CD31	Rat	BD Pharmingen	561410	1/100
PE-Cy7-CD45	Rat	eBioscience	25-0451	1/200
PE-Cy7-PDGFRa (CD140a)	Mouse	Biolegend (Ozyme)	BLE323507	1/50
eFLUOR780<br>(Fixable Viability Dye)		Thermofisher	65-0865	1/1000
Perilipin-2	Rabbit	Novus	NB110-40877SS	1/1000
GAPDH	Mouse	Santa Cruz	sc-32233	1/5000

Secondary Antibody	Host	Coupled with	Supplier	Reference	Dilution
Anti-rat IgG	Donkey	Cy3	Jackson ImmunoResearch	712-166-153	1/1000
		Alexa 647		712-606-153	
Anti-rabbit IgG	Donkey	Cy3	Jackson ImmunoResearch	711-606-152	1/1000
		Alexa 647		711-606-152	
Dapi 1mg/ml			Thermo Scientific	62248	1/2000
APC-Streptavidin			BD Pharmingen	554067	1/200
Anti-mouse BV-421	Rat		BD Biosciences	562580	1/100
Anti-mouse IgG	Donkey	Peroxidase	Jackson ImmunoResearch	715-036-151	1/10000
Anti-rabbit IgG	Donkey	Peroxidase	Jackson ImmunoResearch	711-036-152	1/10000

Supplementary Table 2 – Organoid cell culture media

Medium component	Supplier	Catalogue number	Final concentration
Advanced DMEM/F12	Gibco™	12634010	1x
Glutamax 100x	Gibco™	35050061	1x
Hepes 100x	Gibco™	15630056	1x
Penicillin/Streptomycin 100x	Gibco™	15070063	1x
Wnt3A conditioned medium	Hubrecht Institute	MTA	50% home made
R-spondin conditioned medium	Millipore	SCC111	20% home made
Noggin conditioned medium	Hubrecht Institute	MTA	10% home made
B27 supplement without vitamin A 50x	Gibco™	12587010	1x

N2 supplement 100x	Gibco™	17502048	1x
n-Acetyl Cysteine	Sigma	A9165	1,25 mM
Nicotinamide	Sigma	N0636	10 mM
EGF	Gibco™	PMG8043	50 ng/mL
FGF2	Sigma	SRP4037	50 ng/mL
IGF-1	Biolegend	590906	100 ng/mL
Prostaglandine E2	Tocris	2296	10 nM
Gastrin 1	Sigma	G9145	100 nM
A83-01	Tocris	2939	500 nM
SB202190	Sigma	S7067	3 μM
Primocin 500x	Invivogen	ant-pm-1	1x
LY27632	Sigma	Y0503	10 μM

Supplementary Table 3 – qPCR primer sequences

Gene	Primer sequence
GAPDH	Forward CGTAGACAAAATGGTGAAGGTCGG<br>Reverse AAGCAGTTGGTGGTGCAGGATG
Krt5	Forward GGCCCACAGAGACTGCTTCTTT<br>Reverse AACATTTTGGGGTCTGGGTCAC
Krt17	Forward CTTCCGTACCAAGTTTGAGAC<br>Reverse CGGTTCTTCTCCGCCATCTTC
Krt8	Forward GGACATCGAGATCACCACCT<br>Reverse TGAAGCCAGGGCTAGTGAGT
