# Supplementary material for: A stem cell population at the anorectal junction maintains homeostasis and participates in tissue regeneration
Source: Nat Commun. 2021 May 12;12:2761. doi: 10.1038/s41467-021-23034-x (PMC8115161; doi:10.1038/s41467-021-23034-x)
Supplement: Supplementary file 3 — Reporting Summary [file 41467_2021_23034_MOESM3_ESM.pdf]

nature research

Corresponding author(s): Géraldine Guasch

Last updated by author(s): 2021/02/25

Reporting Summary

Nature Research wishes to improve the reproducibility of the work that we publish. This form provides structure for consistency and transparency in reporting. For further information on Nature Research policies, see our Editorial Policies and the Editorial Policy Checklist.

Statistics

For all statistical analyses, confirm that the following items are present in the figure legend, table legend, main text, or Methods section.

n/a	Confirmed	
[ ]	[x]	The exact sample size (*n*) for each experimental group/condition, given as a discrete number and unit of measurement
[ ]	[x]	A statement on whether measurements were taken from distinct samples or whether the same sample was measured repeatedly
[ ]	[x]	The statistical test(s) used AND whether they are one- or two-sided<br>*Only common tests should be described solely by name; describe more complex techniques in the Methods section.*
[x]	[ ]	A description of all covariates tested
[ ]	[x]	A description of any assumptions or corrections, such as tests of normality and adjustment for multiple comparisons
[ ]	[x]	A full description of the statistical parameters including central tendency (e.g. means) or other basic estimates (e.g. regression coefficient) AND variation (e.g. standard deviation) or associated estimates of uncertainty (e.g. confidence intervals)
[ ]	[x]	For null hypothesis testing, the test statistic (e.g. *F*, *t*, *r*) with confidence intervals, effect sizes, degrees of freedom and *P* value noted<br>*Give P values as exact values whenever suitable.*
[x]	[ ]	For Bayesian analysis, information on the choice of priors and Markov chain Monte Carlo settings
[x]	[ ]	For hierarchical and complex designs, identification of the appropriate level for tests and full reporting of outcomes
[x]	[ ]	Estimates of effect sizes (e.g. Cohen's *d*, Pearson's *r*), indicating how they were calculated

Our web collection on statistics for biologists contains articles on many of the points above.

Software and code

Policy information about availability of computer code

Data collection	Single cells RNA sequencing: Illumina bcl files were basecalled, demultiplexed and aligned to the mouse mm10 genome using the cellranger software (version 3.1.0, 10x Genomics).
Data analysis	ZEN 3.0, Adobe Photoshop CS5, Adobe Illustrator CS5 were used to analyzed images and make figures. ImageJ (Fiji) v1.53 was used to quantify the RNAscope and immunostaining experiments and GraphPad Prism 5.2 to calculate t-test, two-way ANOVA and Bonferroni test. DAVID algorithm (DAVID v6.7) was used to perform a gene functionnal classification. Flow cytometry: FACS ARIA II (for FACS sorting) and FACS Diva software (for FACS data analysis). Single cell RNA sequencing data were analyzed with R/Bioconductor packages (R version 4.0.3), in particular the Seurat package v.3.2.2. Pseudotime analysis were benchmarked with dynverse R package (dynguidelines version 1.0.1) and the slingshot R package (version 1.6.1).

For manuscripts utilizing custom algorithms or software that are central to the research but not yet described in published literature, software must be made available to editors and reviewers. We strongly encourage code deposition in a community repository (e.g. GitHub). See the Nature Research guidelines for submitting code & software for further information.

Data

Policy information about availability of data

All manuscripts must include a data availability statement. This statement should provide the following information, where applicable:

- Accession codes, unique identifiers, or web links for publicly available datasets
- A list of figures that have associated raw data
- A description of any restrictions on data availability

Raw data from the scRNA sequencing have been deposited in the Gene Expression Omnibus under the following codes: GSM4982239 (related to Figure 2 and Extended data figure 5) and GSM4982240 (related to Figure 6 and Extended data figure 8).

https://www.ncbi.nlm.nih.gov/geo/query/acc.cgi?acc=GSE163394
https://www.ncbi.nlm.nih.gov/geo/query/acc.cgi?acc=GSM4982239
https://www.ncbi.nlm.nih.gov/geo/query/acc.cgi?acc=GSM4982240
There are no restrictions on availability of the materials used in the study.

Field-specific reporting

Please select the one below that is the best fit for your research. If you are not sure, read the appropriate sections before making your selection.

☒ Life sciences ☐ Behavioural & social sciences ☐ Ecological, evolutionary & environmental sciences

For a reference copy of the document with all sections, see nature.com/documents/nr-reporting-summary-flat.pdf

Life sciences study design

All studies must disclose on these points even when the disclosure is negative.

Sample size	Samples size for each experiment is written in the corresponding figure legends. For the lineage tracing experiment, the sample size was determined based on previous experiment performed in the laboratory referring to the data on mouse genetic reporter (Runck et al., 2014) and clonogenicity assay (McCauley et al., 2017). For the wound experiment, since we consider that the injury procedure may vary from one mouse to another and since we had no previous experience with this model, we consider that a minimum of ten mice per analysis point was required to yield high power to detect specific effects. No statistical methods were used to predetermine sample size.
Data exclusions	No data were excluded from the analysis
Replication	All the experiments were performed in at least 3 biologically independent replicates and statistics were calculated on biological replicates. All attempts at replication of the results were successful.
Randomization	For in vivo studies on genetic mouse models, animals were chosen based on their correct genotypes requiring two correct alleles (K17CreERT2/R26RGFP). K17CreERT2/GFP mice were treated with Tamoxifen to perform lineage tracing in normal and injured conditions. We minimized the age and sex differences between different animals by using similar numbers of male and female mice at the age of 6-8 weeks after birth. Each experiment contained animals from at least 3 different litters.<br>For cell culture experiments, organoids were derived from at least 3 separate experiments coming from 4 different animals
Blinding	For in vivo studies, the investigators were blinded to mouse genotyping, group allocation during data collection, analysis, imaging and quantification. For cell culture studies, the investigators were blinded to treatment conditions for analysis, imaging and quantification.

Reporting for specific materials, systems and methods

We require information from authors about some types of materials, experimental systems and methods used in many studies. Here, indicate whether each material, system or method listed is relevant to your study. If you are not sure if a list item applies to your research, read the appropriate section before selecting a response.

Materials & experimental systems

n/a	Involved in the study	
☐	☒	Antibodies
☒	☐	Eukaryotic cell lines
☒	☐	Palaeontology and archaeology
☐	☒	Animals and other organisms
☐	☒	Human research participants
☒	☐	Clinical data
☒	☐	Dual use research of concern

Methods

n/a	Involved in the study	
☒	☐	ChIP-seq
☐	☒	Flow cytometry
☒	☐	MRI-based neuroimaging

Antibodies

Antibodies used	All primary and secondary antibodies used are all described in the supplemental table 1 in the manuscript
Validation	The antibodies that are commercially available were validated by the provider. We used the protocols and recommendations of the manufacturer only on validated species. The nectin-4 monoclonal antibody (clone N4.mu1) has been validated by immunofluorescence on Cos cells transfected with an expression vector, carrying the cDNA sequence of murine nectin-4.

Animals and other organisms

Policy information about studies involving animals; ARRIVE guidelines recommended for reporting animal research

Laboratory animals	All animals used are in the C57/BL6J background. Both male and female mice are used in all the experiments. In vivo experiments were performed in accordance with the European Community guidelines for the care and use of laboratory animals. All animal manipulations were performed by qualified personnel having sufficient experience with the described procedures to minimize any possible pain and discomfort to the animals. K17CreERT2/R26RGFP were induced with tamoxifen for two days and sacrificed at different time points for lineage tracing experiments and for FACS-cells isolation when performing the scRNA-sequencing analysis and the organoid experiments.<br>For wounding experiments: 2-4 months old K17CreERT2/R26RGFP mice were injected with tamoxifen two days before the chemical EDTA or mechanical wound. Mice were monitored carefully after the injury and analyzed 1, 2 and 4 weeks post-wound.<br>Sections of prefixed frozen embedded anorectal tissues were used for immunostaining and RNAscope experiments.<br>The housing conditions of all the mice followed strickly the ethical regulation. Mice are housed in individually ventilated cages (IVC, sealsafe plus TECHNIPLAST) according to SPF FELASA standards and food and water were given at libitum. For social enrichment, 6 mice per cages are generally housed with sterile nesting materials such as cotton or compressed wood chips for nidification. The room temperature ranged from 20 and 25°C. The relative ambient humidity was 55% +/-15. Semi-natural light cycle of 12:12 was used.
Wild animals	The study did not involve wild animals
Field-collected samples	The study did not contain field-collected samples
Ethics oversight	All experiments were approved by the European and national regulation (protocols #4572, #8287 and #2244), ethical committee CE14

Note that full information on the approval of the study protocol must also be provided in the manuscript.

Human research participants

Policy information about studies involving human research participants

Population characteristics	Dr. Flora Poizat in the Department of Pathology at the Paoli Calmettes Institute is our coordinator to receive sample under free informed consent. The identity of the participants is not known to scientist in the laboratory. Informed consent files are stored by referring clinicians in a locked place, all data are stored in encrypted and password-locked files. The national rules of data protection are followed.
Recruitment	*Describe how participants were recruited. Outline any potential self-selection bias or other biases that may be present and how these are likely to impact results.*
Ethics oversight	Anorectal tissue samples from adult patients were approved by the Paoli-Calmettes Institute Strategic Orientation Committee (authorization TZ-cancers-IPC 2015-021). Informed written consent was given by the patients. Samples came from discarded tissues after surgery.

Note that full information on the approval of the study protocol must also be provided in the manuscript.

Flow Cytometry

Plots

Confirm that:

- [x] The axis labels state the marker and fluorochrome used (e.g. CD4-FITC).
- [x] The axis scales are clearly visible. Include numbers along axes only for bottom left plot of group (a 'group' is an analysis of identical markers).
- [x] All plots are contour plots with outliers or pseudocolor plots.
- [x] A numerical value for number of cells or percentage (with statistics) is provided.

Methodology

Sample preparation	The anorectal regions of at least five K17CreERT2;R26RGFP mice induced with tamoxifen for 2 days were microdissected under a dissecting scope (Leica MZ6) and pooled in epithelial cell culture medium40 containing 10% Fetal Bovine Serum (FBS) for further dissociation. Tissues were cut in small pieces using scissor and scalpel in 4ml HBSS 1X + 50 µl collagenase 20% from Clostridium histolyticum (Sigma C2674) and were incubated with high agitation 45min at 37°C. Then, 4.2 µl of DNAse I (10mg/ml Sigma DN25) were added followed by 10min incubation at 37°C under agitation. Samples were then resuspended with 12ml cold PBS 1X and placed in a 50ml conical tube filled until 50ml with PBS 1X and centrifuged for 10min at 4°C at 200g. Pellets were resuspended with 20ml cold PBS 1X and filtered through 70µm filter. To avoid cell death, 200µl of FBS was added. To dissociate left over tissues on the filter, 1ml of TrypLE express enzyme (GibcoTM, 12605010) was added followed by 5min incubation at 37°C. Trypsin activity was then stopped by adding 3ml of epithelial cell culture medium +10% FBS and the mix was filtered through 40µm filter and centrifuged 10min at 4°C at 300g. Pellets were resuspended in PBS 1X 2%FBS. Primary antibody was added to samples 30-45min on ice and covered with foil. Samples were then washed with PBS 1X 2%

	FBS and centrifuged 5min at 4°C at 300g. After discarding the supernatant, secondary antibody was added for 20min at 4°C and then washed again with PBS 2%FBS. Finally, FVD780 (1/1000, Thermofisher) was added to stain dying cells.
Instrument	FACS Aria II equipment (BD Bioscience)
Software	FACS DIVA
Cell population abundance	The proportion of anal TZ GFP+ cells in normal condition varied from 0,3% to 1,6% and in wounded condition from 3 to 6%.
Gating strategy	Living cells were selected by forward scatter, side scatter, doublets discrimination and by FVD780 dye exclusion. Epithelial cells were selected based on the expression of EpCam and the exclusion of CD45, CD11b, CD31, PDGFRa.

☒ Tick this box to confirm that a figure exemplifying the gating strategy is provided in the Supplementary Information.
