# Supplementary material for: A stem cell population at the anorectal junction maintains homeostasis and participates in tissue regeneration
Source: Nat Commun. 2021 May 12;12:2761. doi: 10.1038/s41467-021-23034-x (PMC8115161; doi:10.1038/s41467-021-23034-x)
Supplement: Supplementary file 4 — Supplemental_method [file 41467_2021_23034_MOESM4_ESM.pdf]

SUPPLEMENTAL METHODS

A stem cell population at the anorectal junction maintains homeostasis and participates in tissue regeneration

Louciné Mitoyan[1], Véronique Chevrier[1], Hector Hernandez-Vargas[2,3], Alexane Ollivier[1], Zeinab Homayed[4], Julie Pannequin[4], Flora Poizat[5], Cécile De Biasi-Cador[5], Emmanuelle Charafe-Jauffret[1,5], Christophe Ginestier[1], and Géraldine Guasch[1]*

RNAscope quantification was done following the "Guideline on how to quantify RNAscope Fluorescent Assay Results". All images were acquired using a confocal microscope ZEISS LSM 880 with exact same parameters and analyzed with ImageJ.

Analysis:

All channels were projected over the z axis. DAPI and Krt17 were then split. DAPI channel was segmented to create a Mask of the nuclear area. It is in this area that the intensity of the *Krt17* was measured to ensure that the measurement is done within the nuclear area.

To ensure the selection of the DAPI only contained in the epithelium, we used the "Particule analysis". Size of selected objects was set to 200-infinity. With the "Image Calculator" the Mask DAPI was ADDed to mask *Krt17*. ROI (Region Of Interest) of 500 pixels per 500 pixels was created (the same ROI was used for all images), placed and then duplicated. Images were binarized creating a mask of the *Krt17* staining contained in the DAPI Area. The plugin "Extended Particle Analyzer" of the Biovoxxel tool box was used. To ensure the selection of the dots, the analysis parameters were set as 0.01-Infinity for Area (micron^2). This value was established by experimental trials. Segmented particules corresponding to the

dots were created and then this particules were overlayed on the native *Krt17* z projection for parameters measurement in each particules.

<u>Measurement</u>:

We reported measured Area and IntDen (Integrated density) and calculated total Dot number in ROI according to the following formula:

$$\text{Total dot number in ROI} = \frac{Total\ Integrated\ density\ of\ selected\ dots \times Total\ Area}{Average\ Intensity\ per\ Single\ Dot}$$

Data were then normalized on TZ for each condition (see Source Data File).

N.B: The average intensity per single dot was calculated based on the expression of few single dots from 3 different mice and following this formula:

$$\text{Average Intensity per single dot} = \frac{\sum Integrated\ density\ of\ selected\ dots \times \sum Area\ of\ selected\ dots}{Number\ of\ selected\ dots}$$

Legend of supplementary method figure. A representative example of the *Krt17* (red) quantification in different regions (AC, TZ and rectum) quantified in absence (**a**, **b** and **c**) and presence (**d**, **e** and **f**) of tamoxifen (TAM) injected twice and 48h post-wound without TAM (**g**, **h** and **i**). A least 6 areas of each region (anal canal, TZ and rectum) were quantified per mice (n=3 mice per condition). Scale bars are 50µm. Insets for **a**, **b**, **c**, **d**, **e**, **f**, **g**, **h** and **i** are respectively zoom in 2.8 fold, 4.4 fold, 3.4 fold, 1 fold, 5.3 fold, 3.3 fold, 3.6 fold, 4.7 fold and 4.8 fold.

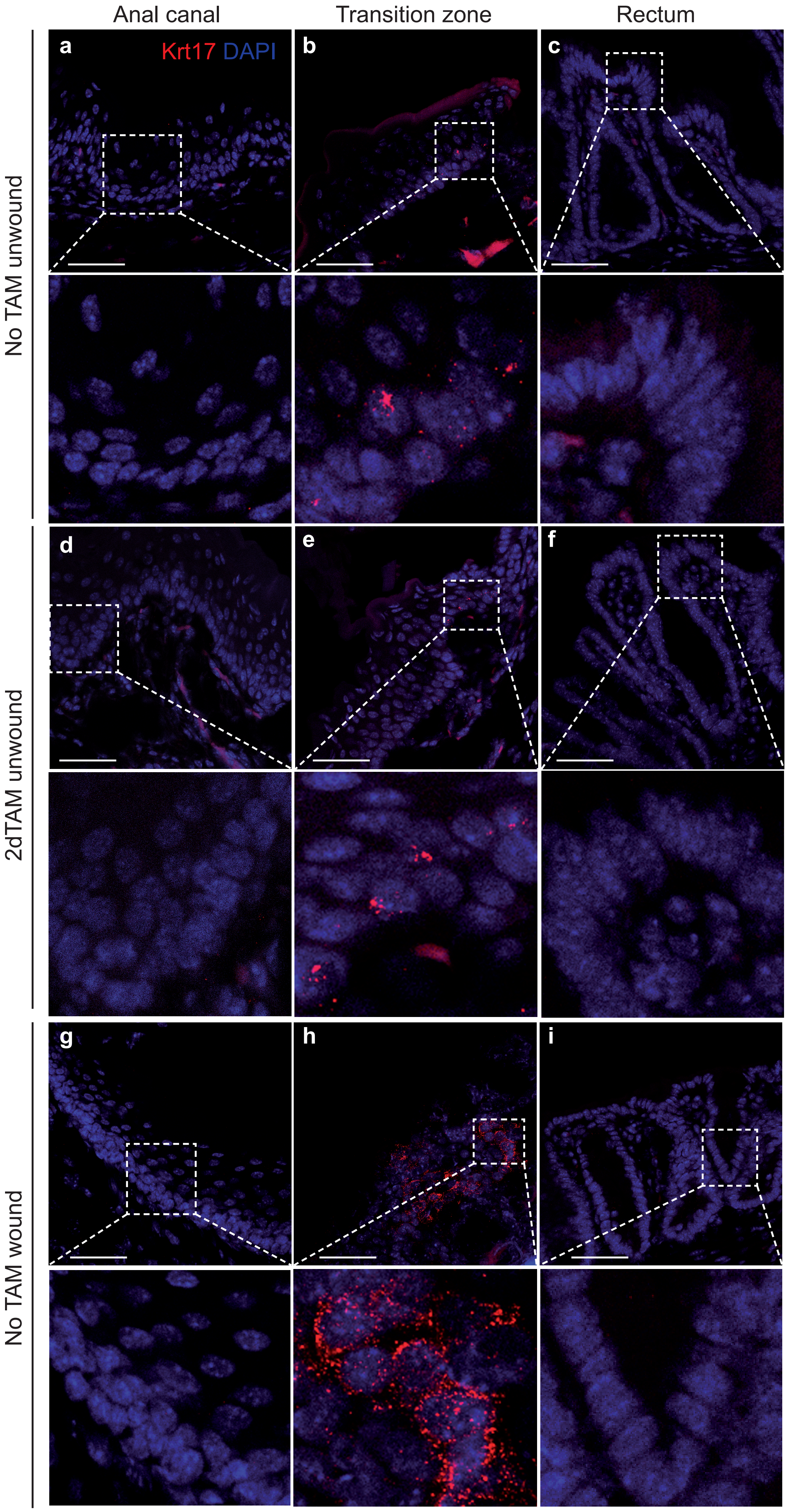
Anal canal
Transition zone
Rectum
a
Krt17 DAPI
b
c
d
e
f
g
h
i
No TAM unwound
2dTAM unwound
No TAM wound
