# Supplementary material for: A stem cell population at the anorectal junction maintains homeostasis and participates in tissue regeneration
Source: Nat Commun. 2021 May 12;12:2761. doi: 10.1038/s41467-021-23034-x (PMC8115161; doi:10.1038/s41467-021-23034-x)
Supplement: Supplementary file 5 — Source Data [file 41467_2021_23034_MOESM5_ESM.zip › Description for source data file.pdf]

Description of Additional Supplementary Files

File Name: Supplementary Data 1
Description: Quantification of the percentage of GFP cells in stratified and glandular tissue (related to Figure 1e)

File Name: Supplementary Data 2
Description: Data source for the quantitative PCR (related to Figure 3c)

File Name: Supplementary Data 3
Description: Full scan Western Blot (related to Figure 3d)

File Name: Supplementary Data 4
Description: Quantification of the percentage of GFP positive cells in glandular epithelium (related to Figure 5d)

File Name: Supplementary Data 5
Description: Full scan Western Blot (related to Figure 6i)

File Name: Supplementary Data 6
Description: Quantification of the percentage of Edu positive cells in TZ and anal canal area (related to Supplementary Figure 1c)

File Name: Supplementary Data 7
Description: Quantification of the *Krt17* RNA expression (related to Supplementary Figure 2c)

File Name: Supplementary Data 8
Description: Quantification of Krt17, Edu and CD34 after tamoxifen injection (related to Supplementary Figure 3)

File Name: Supplementary Data 9
Description: Genes list found in cluster 1, cluster 3 and cluster 7 from single cell RNA sequencing of anorectal cells (related to Supplementary Figure 5)

File Name: Supplementary Data 10
Description: Genes list found in cluster 2, cluster 7 and cluster 8 from single cell RNA sequencing of wounded anorectal cells (related to Supplementary Figure 8)
